# Biological Behaviour and Enhanced Anticorrosive Performance of the Nitrided Superelastic Ti-23Nb-0.7Ta-2Zr-0.5N Alloy

**DOI:** 10.1155/2015/261802

**Published:** 2015-10-25

**Authors:** Valentina Mitran, Cora Vasilescu, Silviu Iulian Drob, Petre Osiceanu, Jose Maria Calderon-Moreno, Mariana-Cristina Tabirca, Doina-Margareta Gordin, Thierry Gloriant, Anisoara Cimpean

**Affiliations:** ^1^Department of Biochemistry and Molecular Biology, University of Bucharest, 91–95 Splaiul Independentei, 050095 Bucharest, Romania; ^2^Institute of Physical Chemistry “Ilie Murgulescu” of Romanian Academy, Splaiul Independentei 202, 060021 Bucharest, Romania; ^3^INSA Rennes, UMR CNRS 6226 Institut des Sciences Chimiques de Rennes, 20 avenue de Buttes de Coëmes, 35708 Rennes Cedex 07, France

## Abstract

The influence of gas nitriding surface treatment on the superelastic Ti-23Nb-0.7Ta-2Zr-0.5N alloy was evaluated. A thorough characterization of bare and nitrided Ti-based alloy and pure Ti was performed in terms of surface film composition and morphology, electrochemical behaviour, and short term osteoblast response. XPS analysis showed that the nitriding treatment strongly influenced the composition (nitrides and oxynitrides) and surface properties both of the substrate and of the bulk alloy. SEM images revealed that the nitrided surface appears as a similar dotted pattern caused by the formation of N-rich domains coexisting with less nitrided domains, while before treatment only topographical features could be observed. All the electrochemical results confirmed the high chemical stability of the nitride and oxynitride coating and the superiority of the applied treatment. The values of the corrosion parameters ascertained the excellent corrosion resistance of the coated alloy in the real functional conditions from the human body. Cell culture experiments with MG63 osteoblasts demonstrated that the studied biomaterials do not elicit any toxic effects and support cell adhesion and enhanced cell proliferation. Altogether, these data indicate that the nitrided Ti-23Nb-0.7Ta-2Zr-0.5N alloy is the most suitable substrate for application in bone implantology.

## 1. Introduction

Titanium (Ti) and its alloys are widely used materials for body implants such as joint prostheses, dental implants, and medical devices because they have high strength-to-weight ratio, excellent corrosion resistance in the* in vivo* physiological environment, and good biocompatibility [1]. However, most commercially available Ti-based implant biomaterials exhibit much higher Young's modulus than that of human bones (around 30 GPa or less). This mismatch of Young's modulus between the Ti-based implant (i.e., 110 GPa for Ti-6Al-4V) and bone is unfavorable for bone healing and remodeling. Thus, a stress shielding effect results in bone resorption and eventually the loosening and premature failure of the implant [2]. Furthermore, the toxicity to human osteoblasts of Al and V ions has been previously pointed out [3]. In addition, the* in vivo* release of Al and V from Ti-6Al-4V alloy due to wear and corrosion has proved to cause neurological disorders and toxicity to human systems, respectively [1, 4]. Therefore, implants alloys with much lower Young's moduli than Ti-6Al-4V and nontoxic elements are being developed. Some examples are Ti-Nb [5], Ti-Nb-Ta [6–8], Ti-Nb-Zr [9–11], Ti-Nb-Mo [12], Ti-Nb-Sn [13], Ti-Nb-N(O) [14, 15], Ti-Nb-Zr-Sn [16–18], Ti-Nb-Mo-Sn [19], Ti-Nb-Mo-Zr-Sn [20], and Ti-Zr-Nb-Fe [21].

Despite their good properties suitable for biomedical applications, Ti-based alloys suffer from the drawback of poor tribological properties like low abrasive and adhesive wear resistance, poor surface hardness, and high coefficient of friction [22]. A number of studies have been performed to resolve these problems. Thus, several forms of nitriding have been developed in order to improve surface properties and bioperformance of Ti-based biomaterials. Some of these comprise gas nitriding [23–26], laser nitriding [27–29], and several forms of plasma nitriding [30–33]. The high temperature gas nitriding technique holds great potential for biomedical applications. Thus, it presents the advantage to coat complex shaped surfaces, which is particularly interesting in the case of implants and prostheses [22]. This technique is also particularly well adapted with the *β* Ti alloys as this microstructure is stable at high temperature. On the other hand, the presence of internal nitrides, observed in the *β*-type titanium alloys, has recently been shown to enhance the adherence to the coating, which is required to avoid the dissemination of wear debris from implant [34].

It is worth mentioning that the studies on the antibacterial activity and biological performance of nitrided Ti-based alloys are rarely reported. Some of them emphasized that by nitriding processes the biomaterial surfaces are endowed with antibacterial properties in terms of bacterial adhesion and biofilm formation [35–38]. Apart from this, the biocompatibility and high corrosion resistance in the physiological environment make nitrided biomaterials a very good choice for load-bearing implants. The physiological fluid contains very aggressive Cl^−^ ions which can corrode the implant materials by releasing ions of the metallic constituents. This dissolution process can affect the implant mechanical properties and can contaminate the organism with metallic ions and compounds. Therefore, an important restriction for a good material is its high corrosion resistance, high stability, and chemical inertness in the human biofluids [37, 39–42]. The nitride coatings can confer high corrosion protection and wear resistance to the implant alloys [43–45], maintaining good mechanical properties and biocompatibility [43, 46]. Huang et al. [47] reported that TiN film was deposited onto the Ti substrate using the cathodic arc plasma deposition technique and ion-nitriding treatment improved corrosion resistance and osteoblast-like cell adhesion. Another study pointed out that TiN coating of Ti plasma sprayed implants exhibited a good affinity for primary human bone marrow mesenchymal stem cells in terms of adhesion, proliferation, and osteogenic differentiation. Jang et al. [48] investigated the effect of TiN and TiAlN produced by physical vapor deposition on Ti dental implants upon MG63 cell behaviour. The analysis of the osteoblast proliferation and differentiation indicated a high level of biocompatibility of the treated Ti dental implant.

To the best of our knowledge there is no literature available on nitrided *β*-type superelastic Ti alloys. In this context, the present study aims to investigate the influence of gas nitriding process on the surface film composition and morphology, corrosion behaviour, and biocompatibility of recently developed Ti-23Nb-07Ta-2Zr-0.5N alloy. The obtained data were related to the properties displayed by the corresponding bare alloy and pure Ti as a reference biomedical implant.

## 2. Experimental

### 2.1. Alloy Synthesis and Superficial Nitriding Process

As titanium, zirconium, tantalum, and niobium have melting points and densities which are very different, the synthesis of the Ti-23Nb-07Ta-2Zr-0.5N alloy composition (mol%) was realized by cold crucible semilevitation melting (CCLM) technique under high vacuum, using a high-frequency magnetic induction generator heating system. With this method, the high-frequency magnetic field is used to stir the liquid, which is useful in ensuring that alloying elements are fully mixed into the melt without contamination thanks to the restricting contact points between the melt and the cold crucible. The added elements are pure solid metals, except nitrogen, which was introduced through titanium nitride (TiN) powder. After a homogenization treatment at 950°C for 16 h, the ingot was cold rolled at room temperature to reach 1 mm in thickness that corresponds to 90% of reduction in thickness.

From the sheet, disc shape samples (diameter: 13 mm, thickness: 1 mm) were cut for the biological and the electrochemical tests. Then, all samples were solution treated under high vacuum at 850°C for 0.5 hour in the beta-phase field and water quenched. The aim of this treatment is to restore a fully recrystallized beta microstructure from the cold rolled state that gives a low elastic modulus (50 GPa) and a superelastic behaviour (2.2% of elastic recovery obtained by tensile test) as previously reported in a recent work [49]. Finally, all samples were mechanically polished on silicon carbide abrasive papers (up to 4000 grit) and then ultrasonically cleaned in acetone, thoroughly washed with ethanol and dried in air. The roughness value in surface, Ra, was evaluated to be 98 ± 7 nm by atomic force microscopy (AFM).

A part of the disc shape samples was superficially nitrided in this study in order to test the biological and the electrochemical responses by comparison with the bare alloy. The nitriding process consists of a high temperature gas nitriding treatment, which was carried out at 950°C for 2 h under one controlled flow high-purity nitrogen atmosphere (N2 > 99.99%).

### 2.2. Evaluation of Composition and Morphology of the Initial Films Existing on Bare and Nitrided Ti-23Nb-0.7Ta-2Zr-0.5N Alloy Surface

The compositions of the native passive film on the bare Ti-23Nb-0.7Ta-2Zr-0.5N alloy surface and of the coating formed on the nitrided alloy surface were identified by X-ray photoelectron spectroscopy (XPS). The surface morphology was assessed by scanning electron microscopy (SEM).

XPS equipment has a source of Alk*α* radiation (1486.6 eV, monochromatized) and the overall energy resolution of 0.65 eV by the full width at half maximum (FWHM) of Au 4f_7/2_ line. The errors in the quantitative analysis (relative concentration) were estimated in a range of ±5% and the accuracy of the binding energies assignments was ±0.2 eV. The thickness of the surface layers was determined by the XPS depth profiling, layer by layer experiment, using Ar^+^ ion beam as an incident angle of 45°, with a spot size of 100 *μ*m, on area of 3 × 3 mm.

SEM micrographs were obtained in a FEI Quanta 3D FEG Dual Beam apparatus operating at accelerating voltage of 20 kV, equipped with backscattered (BSE) detector and energy dispersive X-ray (EDX) spectrometer.

### 2.3. Evaluation of the Electrochemical Behaviour of Ti and Bare and Nitrided Ti-23Nb-0.7Ta-2Zr-0.5N Alloy in Ringer's Solution of Different pH Values

The electrochemical behaviour of Ti and bare and nitrided Ti-23Nb-0.7Ta-2Zr-0.5N alloy in Ringer's solution of different pH values was studied by the potentiodynamic cyclic and linear polarization tests. The measurements were performed with Voltalab 80 equipment.

Cyclic potentiodynamic polarization curves were recorded starting from a value of about 300 mV more negative than the open circuit potential and continue to positive direction till +1000 mV (versus SCE) with a scan arte of 1 mV/s. The main electrochemical parameters were determined: *E*_corr_: corrosion potential as zero current potential; *E*_*p*_: passivation potential where the current density becomes constant; |*E*_corr_ − *E*_*p*_|: tendency to passivation with low values characterizes a strong, easy passivation; *i*_*p*_: passive current density representing the corrosion resistance of the passive layer [37, 44, 45, 50].

Linear potentiodynamic polarization was carried out for ±50 mV around the open circuit potential at a scan rate of 0.1 mV. Tafel representations [42, 51–53] were adjusted by VoltaMaster 4 program and the main corrosion parameters were directly supplied: *i*_corr_: corrosion current density; *V*_corr_: corrosion rate; *R*_*p*_: polarization resistance. Because the polarization resistance, *R*_*p*_, shows the protection degree of the coating [54], it is used to calculate the porosity factor *P*(%) by Tato and Landolt [55] formula:

(1) $$\begin{array}{c} P\left(\;\%\;\right)=\frac{R_{b}}{R_{n}}\times 100, \end{array}$$

where *R*_*b*_ and *R*_*n*_ represent the resistance of the bare and nitrided alloy, respectively. The protection efficiency, *E*(%), of the coating was calculated by many authors [40, 50] in function of the corrosion current density for bare, *i*_corr,*b*_, and nitrided, *i*_corr,*n*_, alloys:

(2) $$\begin{array}{c} E\left(\;\%\;\right)=\frac{i_{\text{corr},b}-i_{\text{corr},n}}{i_{\text{corr},b}}\times 100. \end{array}$$

Also, using the corrosion rate we calculated the ion release rate representing the total quantity of ions released into surrounding biofluid [52, 53]:

(3) $$\begin{array}{c} \text{Ion release rate}=1.016\times V_{\text{corr}}\times 10^{2}, \end{array}$$

where ion release rate is expressed in ng/cm^2^ and *V*_corr_ in *μ*m/year.

The electrochemical experiments took place in a glass electrochemical cell; discs of Ti and bare and nitrided Ti-23Nb-0.7Ta-2Zr-0.5N alloy were used as the working electrodes connected with saturated calomel electrode (SCE) by a Luggin-Haber capillary; auxiliary electrode was a platinum plate. The working electrodes were ultrasonically degreased in acetone and bidistilled water for every 30 min and dried in air. Three samples were used for every experiment and the results reproducibility was very good.

The real conditions from the human body were simulated by Ringer's solution of different pH values: acid pH of about 3 value [56] can appear in the case of surgery; neutral pH of about 7.4 value is the normal pH of the human biofluid [56, 57]; alkaline pH of about 9 value [57] can arise in case of infections or inflammations. Ringer's solution composition (g/L) was NaCl, 6.8; KCl, 0.4; CaCl_2_, 0.2; MgSO_4_·7H_2_O, 0.2048; NaH_2_PO_4_·H_2_O, 0.1438; NaHCO_3_, 1.1; glucose, 1.

### 2.4. Culture Model and Cell Cultivation

The cell culture model used in cytocompatibility studies was represented by a human osteoblast-like cell line, MG63 (American Type Culture Collection, CRL-1427). These cells were seeded onto the test materials at a final density of 5 × 10^3^ cells·cm^−2^ in Dulbecco's Modified Eagle Medium (DMEM) containing 1‰ glucose, supplemented with 10% heat-inactivated fetal bovine serum and 1% (v/v) penicillin/streptomycin (10,000 units·mL^−1^ penicillin and 10 mg·mL^−1^ streptomycin). Previously to* in vitro* tests, the Ti-based samples were sterilized by immersion in 70% ethanol, washed with sterile-filtered Milli-Q water, maintained under ultraviolet light in a sterile tissue culture hood for 1 h on each side, and conditioned in culture medium. The cells were maintained in contact with these samples in a humidified atmosphere of 5% CO_2_ at 37°C for specific points in time. The medium was exchanged every second day. All experiments have been done in triplicate and a statistical analysis has been performed using one-way ANOVA with Bonferroni's multiple comparison tests.

### 2.5. Osteoblasts Adhesion and Morphology

MG63 osteoblast-like cells grown on Ti-based materials for 30 min, 6 h and 24 h, were fixed with 4% paraformaldehyde, permeabilized and blocked with 0.1% Triton X-100/2% bovine serum albumin for 1 h and, subsequently, washed with phosphate buffered saline (PBS). The samples were then incubated with anti-vinculin antibody (dilution 1 : 50, Santa Cruz Biotechnology) for 2 h at room temperature, washed again with PBS, and incubated for 1 h with a specific secondary antibody coupled with Alexa Fluor 546. Afterwards, a sequential treatment with Alexa Fluor 488 conjugated to phalloidin and 2 *μ*g/mL DAPI (4′,6-diamidino-2-phenylindole) was performed. Labeled samples were washed with PBS and examined under an inverted microscope equipped with epifluorescence (Olympus IX71). The images were captured by means of Cell F image acquiring system.

### 2.6. Cell Viability and Proliferation

Viability of MG63 osteoblasts cells was evaluated by combining a cytotoxicity test consisting in the assessment of lactate dehydrogenase (LDH) released into the culture medium and MTT [3-(4,5-dimethyl thiazol-2-yl) 2,5-diphenyltetrazolium bromide] colorimetric study which also represents a useful indication of cell proliferation rates. LDH assay was carried out by using a cytotoxicity detection kit (Tox-7, Sigma-Aldrich) according to the manufacturer's protocol. Absorbance was evaluated at 490 nm using a microplate reader (Thermo Scientific Appliskan). MTT assay has been performed as previously reported [58]. Both assays were conducted at 1, 3, and 5 days after seeding.

### 2.7. Statistical Analysis

Statistical analysis was performed with GraphPad Prism software using one-way ANOVA with Bonferroni's multiple comparison tests. Triplicate samples were used in LDH and MTT experiments to ensure the reproducibility of the results. The data are presented as means ± SD (standard deviation). The *p* values <0.05 were considered to be statistically significant.

## 3. Results and Discussion

In order to demonstrate the advantage of surface gas nitriding of the recently developed *β*-type Ti alloy, Ti-23Nb-0.7Ta-2Zr-0.5N, the surface film composition and morphology, corrosion behaviour, and* in vitro* osteoblast response have been assessed in this study. In a previous paper, this superelastic alloy combining high strength, high superelasticity, low Young's modulus (approximately 50 GPa), and good ductility was shown to hold a great potential for orthopedic applications [49]. Thus, a substantial increase in the differentiation and mineralization ability of MC3T3-E1 preosteoblasts and no significant inflammatory response were elicited in cells cultured on this material.

### 3.1. Composition and Morphology of the Initial Films Existing on the Bare and Nitrided Ti-23Nb-0.7Ta-2Zr-0.5N Alloy Surfaces

#### 3.1.1. Composition of the Initial Films Existing on the Bare and Nitrided Ti-23Nb-0.7Ta-2Zr-0.5N Alloy Surfaces from XPS Analysis

In order to remove the unavoidable surface carbon contamination originated from environmentally hydrocarbon adsorption, a gentle Ar^+^ ion etching of 0.5 min sputtering with a beam energy of 1 keV scanned over (3 × 3) mm area was carried out. XPS experiments performed under this setup on standard samples of Ti, Nb, Ta, and Zr oxides showed no ion beam induced effects on their surface chemistry.

**Figure 1:**
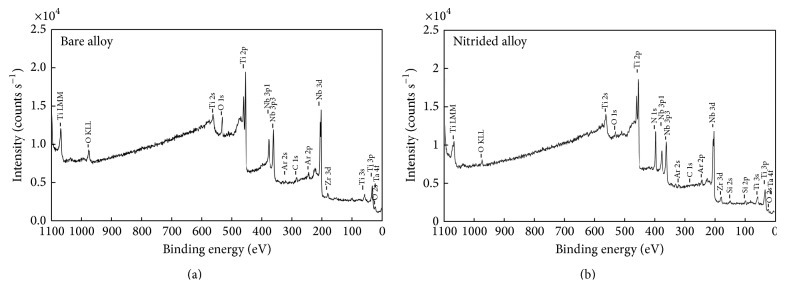
XPS survey spectra for bare (a) and nitrided (b) Ti-23Nb-0.7Ta-2Zr-0.5N alloy.

XPS survey spectra for the native passive film existing on the bare Ti-23Nb-0.7Ta-2Zr-0.5N alloy (Figure 1(a)) detected the presence of the characteristic peaks for all constituent elements Ti 2p, Nb 3d, Zr 3d, Ta 4f, O 1s, and N 1s [59, 60]. The same elements were identified in XPS survey spectrum for the nitrided Ti-23Nb-0.7Ta-2Zr-0.5N alloy (Figure 1(b)) with the difference that the peak for N 1s is much more intense (due to a massive quantity of nitrogen from the nitride coating) and the peak for O 1s is less intense compared with spectrum for the bare alloy. The thickness of the native passive film existing on the bare Ti-23Nb-0.7Ta-2Zr-0.5N alloy surface is 6.5 ± 0.5 nm calculated by using the relative sputter rates experimentally determined by Baer et al. [61] on the same equipment and the same experimental setup. For the nitrided Ti-23Nb-0.7Ta-2Zr-0.5N alloy, the thickness of the formed layer could not be determined because it exceeds the detection limits of our equipment as it is a very thick coating.

**Figure 2:**
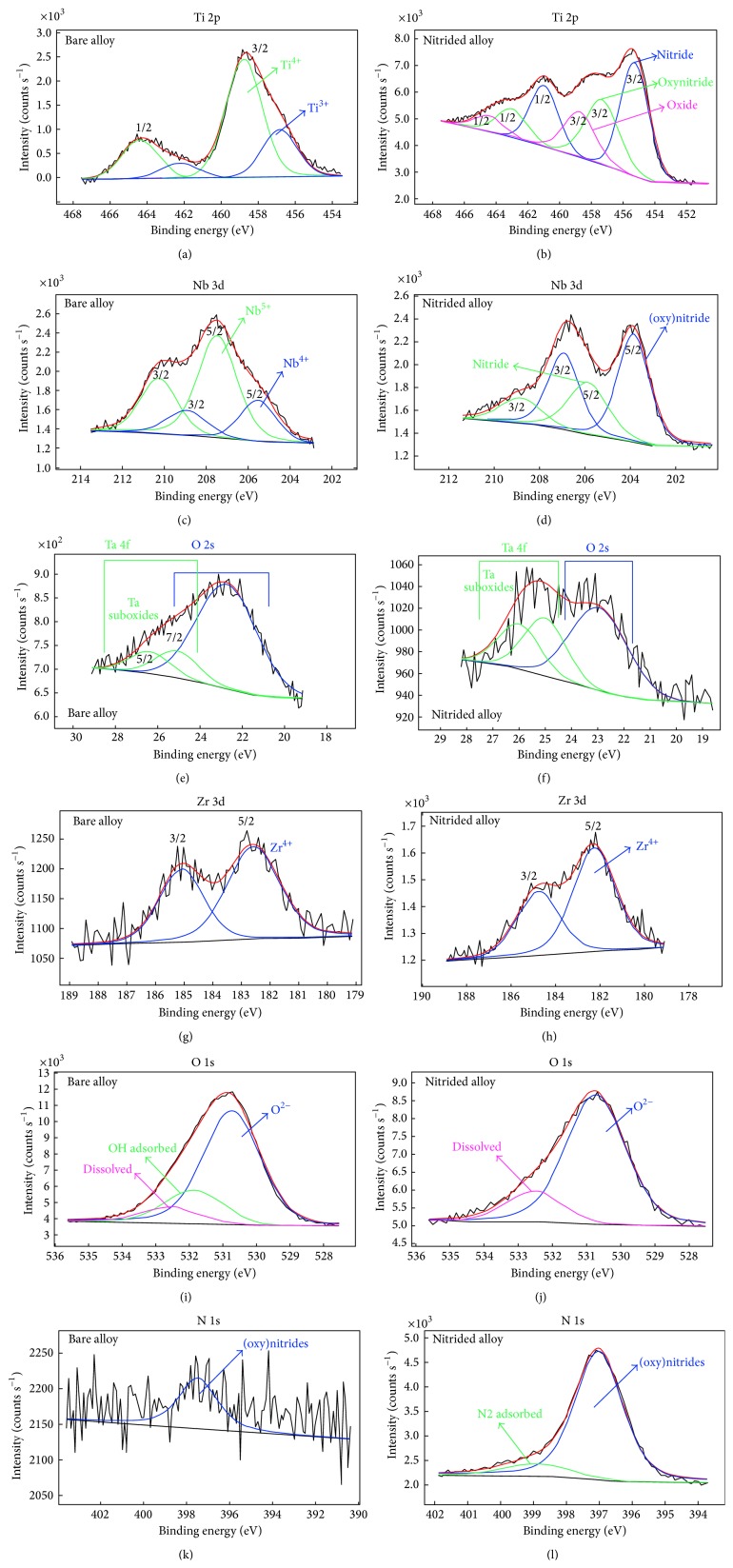
XPS high resolution spectra: (a) Ti 2p for bare alloy; (b) Ti 2p for nitrided alloy; (c) Nb 3d for bare alloy; (d) Nb 3d for nitrided alloy; (e) Ta 4f for bare alloy; (f) Ta 4f for nitrided alloy; (g) Zr 3d for bare alloy; (h) Zr 3d for nitrided alloy; (i) O 1s for bare alloy; (j) O 1s for nitrided alloy; (k) N 1s for bare alloy; (l) N 1s for nitrided alloy.

The XPS high resolution spectra (Figure 2) differ very much referring to the bare and nitrided Ti-23Nb-0.7Ta-2Zr-0.5N alloy.

Ti 2p for the bare alloy (Figure 2(a)) is present as a mixture of 4+ oxidation state and 3+ oxidation state as well as a tiny amount of TiN; nitrogen is found at the limit of detection of our XPS equipment (~0.5 atom %). It is worth to notice that the only photoelectric emission method allows the detection of nitrogen at this low limit; otherwise, no other method has the capability to detect and to resolve nitrogen characteristic feature in a matrix with oxygen and suboxides.

Ti 2p for the nitrided alloy (Figure 2(b)) evidences the peak for TiO_2_ oxide and supplementary peaks for the TiN and oxynitride.

Nb 3d for the bare alloy (Figure 2(c)) shows a mixture of Nb_2_O_5_ and NbO_2_ oxides.

In Nb 3d deconvoluted spectra for the nitrided alloy (Figure 2(d)) niobium oxides did not appear; only niobium nitride and oxynitride did.

Photoelectron Ta 4f doublet lines are very close to O 2s line; however, the deconvoluted procedure for the bare alloy (Figure 2(e)) exhibited the presence of tantalum suboxides on the outermost surface layer after removing the contaminated top surface layer.

In the case of the nitrided alloy, Ta 4f (Figure 2(f)) is maintained as suboxides in very low concentration.

Zr 3d for the bare alloy (Figure 2(g)) shows a tendency to forming only 4+ oxidation state (ZrO_2_) without any suboxides.

The same peak for the protective ZrO_2_ oxide was revealed for the nitrided alloy (Figure 2(h)) proving a high stability in its full oxidation state.

O 1s spectrum for the bare alloy (Figure 2(i)) depicts three peaks, three features: the oxygen (O^2−^) bonded into the lattice as oxides; hydroxyl group (OH^−^) adsorbed from the environment; and a small amount of oxygen dissolved into the material matrix.

For the nitrided alloy, O 1s spectrum (Figure 2(j)) does not display the peak for the adsorbed OH^−^ group.

N 1s deconvoluted spectrum for the bare alloy (Figure 2(k)) displays a low content of nitrogen at the detection limit of our instrument (~0.5% atom) assigned to the (oxy)nitride chemical state. The presence of a tiny amount of adsorbed nitrogen cannot be completely ruled out.

For the nitrided alloy, N 1s spectrum indicates peaks for nitrides, oxynitrides, and adsorbed nitrogen. The massive presence of the nitrogen that reacts with the surface and with the bulk of the alloy, till depth in the range of microns, diminishing the thickness of the native passive film under 5 nm was revealed. It is clear that, in the region where the oxygen is present, nitrides and oxynitrides are formed; then, after oxygen consumption, only “in-depth” nitrides are formed. Oxygen has a lower concentration correlated to the much higher nitrogen atom concentration if compared with the bare alloy. Thus, the nitriding treatment strongly influences both the composition and properties of the surface, of the substrate and of the bulk alloy.

Atom relative concentrations of the initial and nitrided film on Ti-23Nb-0.7Ta-2Zr-0.5N alloy surface are displayed in Table 1.

#### 3.1.2. Morphology of the Initial Films Existing on the Bare and Nitrided Ti-23Nb-0.7Ta-2Zr-0.5N Alloy Surfaces from SEM Micrographs

**Figure 3:**
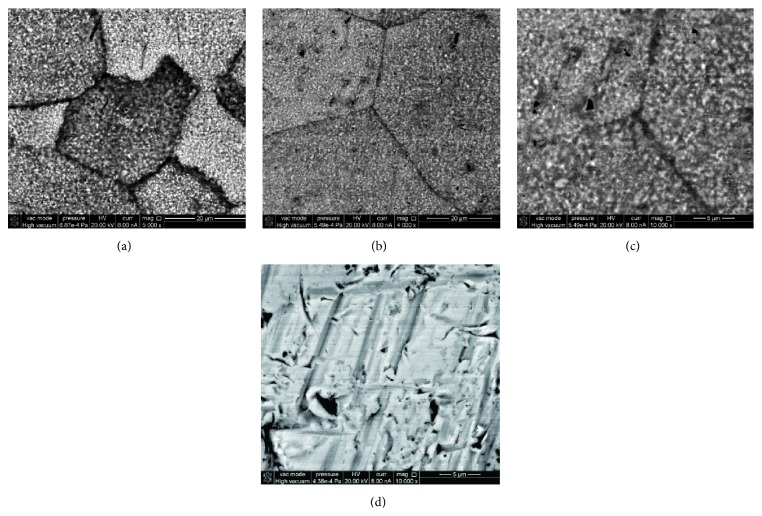
SEM images spectra for nitrided (a–c) and bare (d) Ti-23Nb-0.7Ta-2Zr-0.5N alloy in BSE mode.

The alloy surface after nitration shows an etched-like texture, with the grain boundaries preferentially affected by the treatment, due to their higher reactivity (Figures 3(a) and 3(b)). The backscattered electron (BSE) images show dark grain boundaries due to a higher content of N (Figures 3(a)–3(c)). The thickness of the dark grain boundaries ranges between 200 nm and around 1 *μ*m, indicating that the N has penetrated those lengths inside the alloy grains. Apart from the preferential nitration of the grain boundaries, it is clear that the treatment has significantly changed the whole surface of the alloy. The nitrided surface of each grain appears with a similar dotted pattern. The dotted pattern is caused by the formation of N-rich domains coexisting with less nitrided domains, visible as brighter spots in the images, while before treatment (Figure 3(d)) only topographical features could be observed, neither grain boundaries nor the presence of domains inside the grains.

**Figure 4:**
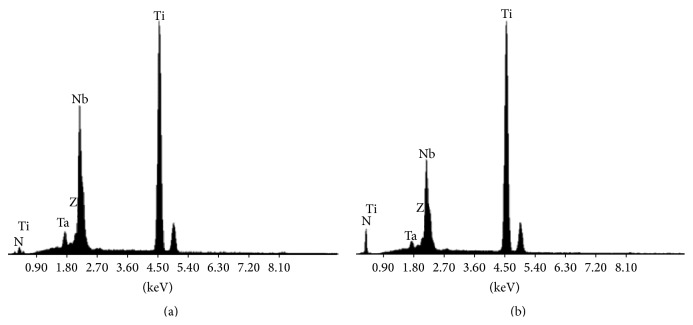
EDX spectra for bare (a) and nitrided (b) Ti-23Nb-0.7Ta-2Zr-0.5N alloy.

The EDX elemental analysis (Figure 4) detected the alloy elements and nitrogen, with a minor presence of N in the original alloy, clearly growing up after nitration to about 33 at.%, according to the EDX quantitative analysis, a value in line although slightly lower than what is given by XPS measurements. We estimate that the electron penetration depth at 20 keV, and therefore the depth of the region measured by EDX, exceeds one micron, explaining the very weak contribution of O from the very thin oxide passivation layer in the original alloy. Besides EDX measurements confirm the nitridation of the surface to a micron-scale depth and also indicate that the decrease of Nb content detected by XPS measurements at the outer surface of the nitrided alloy is characteristic of the whole nitrided layer.

### 3.2. Electrochemical and Corrosion Behaviour of Bare and Nitrided Alloy in Comparison with Ti

The electrochemical behaviour of the bare and nitrided alloy in comparison with Ti in Ringer solution was appreciated from the cyclic potentiodynamic polarization curves and main electrochemical parameters. The corrosion resistance was evaluated from Tafel representations by the main corrosion parameters.

#### 3.2.1. Electrochemical Behaviour of Bare and Nitrided Alloy in Comparison with Ti

**Figure 5:**
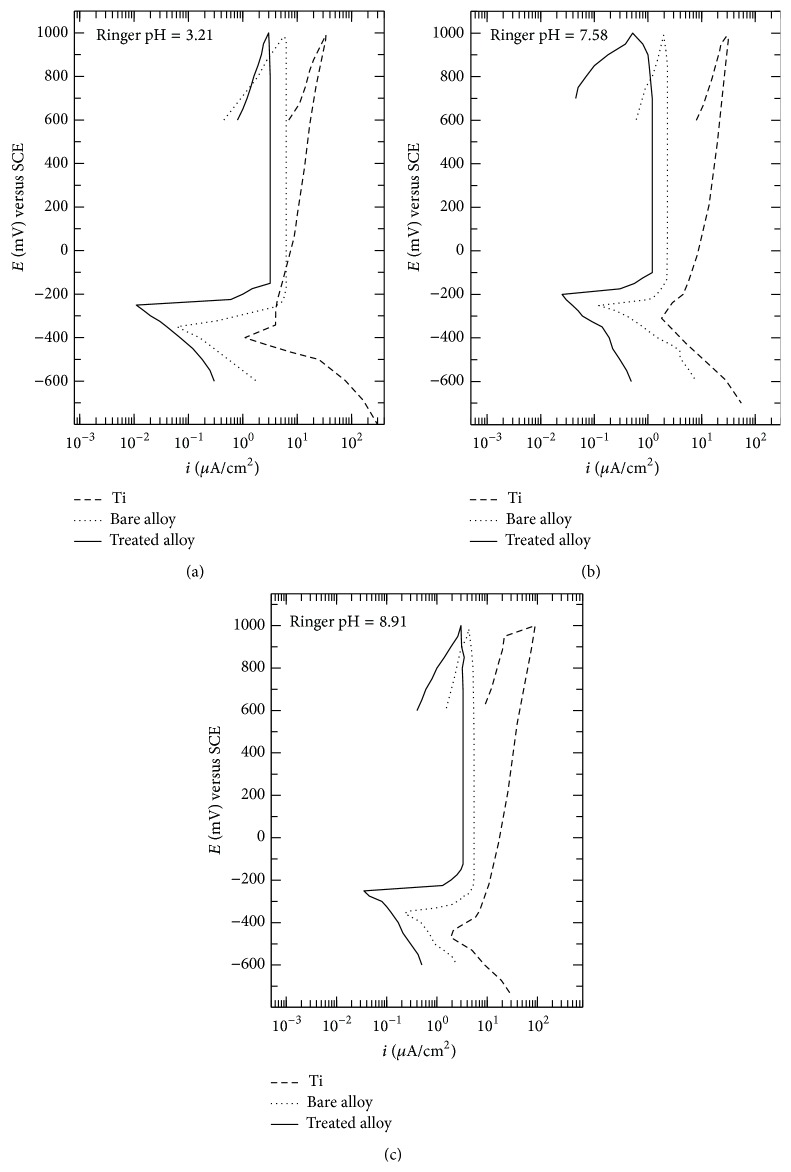
Cyclic potentiodynamic polarization curves for Ti and bare and nitrided Ti-23Nb-0.7Ta-2Zr-0.5N alloy in Ringer solution of different pH values, at 37°C.

Polarization curves (Figure 5) of the bare and treated alloy in Ringer solution shifted to left and to more positive potentials showing an ennobling of the corrosion, *E*_corr_, and passivation, *E*_*p*_, potentials and a reduction of the passive current density, *i*_*p*_; the *i*_*p*_ values are constantly maintained on the whole passive potential range, indicating the continuous thickening of the protective layers only by the ionic transfer inside these layers, in concordance with the high field mechanism [62].

From Table 2 the following can be observed.

*E*_corr_ and *E*_*p*_ values for the treated alloy are about 150 mV more electropositive than those of Ti and about 100 mV more electropositive than those of the bare alloy, denoting the enhancement of the corrosion resistance due to the protective action of the nitride and oxynitride coating [39, 45, 51].

|*E*_corr_ − *E*_*p*_| values for the nitrided alloy are lower compared with Ti and the bare alloy, which means a stronger, more rapid, easier passivation as a result of the contribution of the nitride and oxynitride coating that acts as a barrier layer against the ion crossing through it [40, 45].

All electrochemical results confirmed the high chemical stability of the nitride and oxynitride coating and the superiority of the applied treatment.

#### 3.2.2. Corrosion Resistance of Bare and Nitrided Alloy in Comparison with Ti

From the corrosion parameters (Table 3) the following observations can be summarized:

 (i) the values of the corrosion current density, *i*_corr_, corrosion rate, *V*_corr_, and ion release rate for the processed alloy significantly decreased in comparison with those of Ti and bare alloy, namely, a very high corrosion resistance that attests a denser, very stable coating [40, 44, 45];
(ii)  the polarization resistance, *R*_*p*_, of the treated alloy increased with about three orders of magnitude, with respect of the untreated alloy, a fact that proves the high chemical stability and the inertness of the nitride and oxynitride coating [37, 39, 45];
(iii)  the protective efficiency factor, *E*, has very high values, over 91%, reflecting the very good protective action of the applied coating, due to its adhesion and compactness that comprises both nitrides and oxynitrides [51] (by XPS analysis);
(iv)  in the same time, the porosity factor, *P*, has moderate values around 32–36%, in relation with SEM images.

The values of the corrosion parameters ascertained the excellent corrosion resistance of the coated alloy in the real functional conditions from the human body, namely, Ringer's solution of different pH values.

### 3.3. *In Vitro* Behaviour of Human Osteoblasts

It has to be highlighted that very few studies addressed the* in vitro* biocompatibility of the superelastic Ni- free Ti-based alloys [21, 49, 58, 63] and there is no available literature on their* in vivo* osseointegration capacity. In a previous study [58], our group established that the superelastic Ti-25Nb-25Ta and the commonly employed implant material, Ti-6Al-4V, exhibited almost equivalent hFOB 1.19 osteoblast response in terms of viability, cell attachment and spreading, morphological behaviour, production of fibronectin and its organization into extracellular network, and cell proliferation potential [58]. Park et al. [63] assessed the MC3T3-E1 preosteoblast response to the ultrafine-grained (UFG) and coarse-grain (CG) Ti-13Nb-13Zr alloy as compared to CG Ti-6Al-4V. The experimental results conducted to the conclusion that the UFG and CG Ti-13Nb-13Zr alloys exhibit significantly increased cellular attachment compared with CG Ti-6Al-4V alloy. Furthermore, the analyzed differentiation markers showed the highest levels of expression on the superelastic Ti alloys, especially on the ultrafine-grained one. In another study, a novel superelastic Ti-19Zr-10Nb-1Fe alloy with low Young's modulus (59 GPa) was fabricated [21]. The* in vitro* experiments with MC3T3-E1 preosteoblasts demonstrated almost similar extent of cell adhesion and proliferation on both the Ti-19Zr-10Nb-1Fe and NiTi alloys.

**Figure 6:**
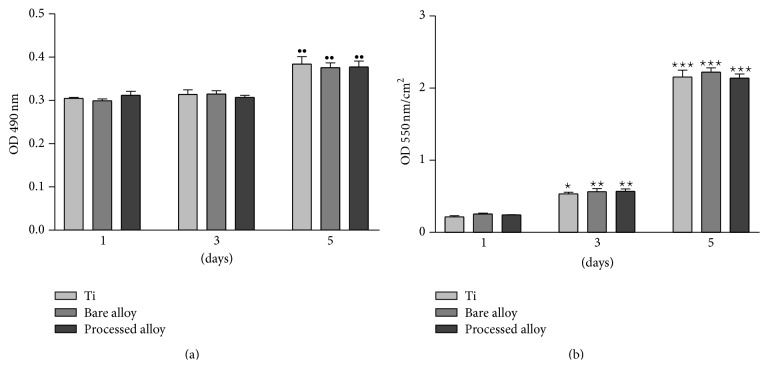
Viability of MG63 osteoblast-like cells cultured onto Ti and bare and nitrided Ti-23Nb-0.7Ta-2Zr-0.5N alloy for 1, 3, and 5 days as determined by (a) LDH and (b) MTT assays. Data analysis was based on mean ± SD (*n* = 3). ^••^ *p* < 0.01 versus corresponding sample at 1 and 3 days; ^⋆^ *p* < 0.05 versus corresponding sample at 1 day; ^⋆⋆^ *p* < 0.01 versus corresponding sample at 1 day; ^⋆⋆⋆^ *p* < 0.001 versus corresponding sample at 1 day.

In this context, the aim of our biological assays was to comparatively evaluate the* in vitro* behaviour of human osteoblast-like cells MG63 on bare and nitrided superelastic *β*-type Ti-23Nb-07Ta-2Zr-0.5N alloy and Ti. An important feature of any biomaterial for clinical applications is the lack of cytotoxicity. In order to determine the possible detrimental effects exerted by all biomaterials on the cell viability, the release of LDH into the culture media was quantified as an index of cell death. As it can be seen in Figure 6(a), MG63 cells exhibit a comparable viability. Thus, the time-dependent profiles of LDH activity revealed no significant differences between the three analyzed substrates at any point in time. Moreover, LDH activity recorded approximately similar optical density (OD) values at 1 and 3 days after seeding. A higher activity but not a significant one for the loss of membrane integrity was remarked between the third and fifth days of culture. This could be associated with the confluence-initiated cell death. Consequently, all three samples elicit no significant cytotoxic responses from MG63 osteoblasts and do not alter cell viability. Furthermore, the proliferation status of the total population of cells was assessed by MTT colorimetric method. As shown in Figure 6(b), the number of viable metabolically active osteoblasts on the analyzed substrates equally increased from the first to the fifth day of culture. By day 5, the proliferation rates on the three surfaces were significantly higher than those found after 1 and 3 days of culture. Taken together, these findings prove that all studied biomaterials favor cell proliferation without showing any deleterious effect suggesting increased biocompatibility.

**Figure 7:**
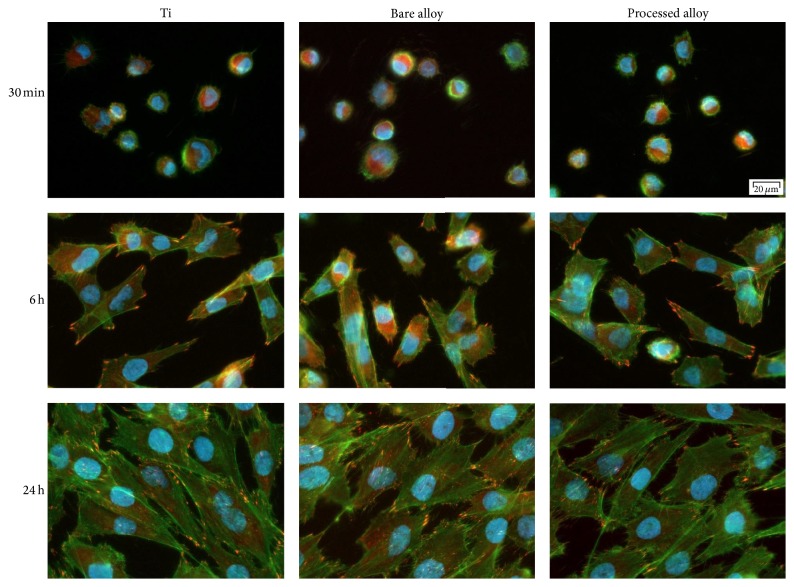
Merged fluorescence images of actin filaments (green) and vinculin (red) in MG63 osteoblast-like cells grown on Ti and bare and nitrided Ti-23Nb-0.7Ta-2Zr-0.5N surfaces. The nuclei are stained in blue with DAPI. Scale bar represents 20 *μ*m.

Cells attach to the material surface by a variety of cellular microextensions, such as filopodia and lamellipodia. In this study, cell-to-substrate interaction was observed by double fluorescent staining of vinculin and actin filaments. It is largely accepted that the initial interaction of cells with the biomaterial will influence their further fate. Consequently, cell adhesion and morphology were both investigated in order to assess the biocompatibility of the nitrided Ti-23Nb-0.7Ta-2Zr-0.5N substrate as compared to the bare one and Ti reference biomaterial. A major protein of focal adhesions and a key player in the regulation of cell adhesion is vinculin [64]. Hence, vinculin provides a valuable detection system for focal adhesion sites by means of specific antibody detection. In this study, after 30 min of culture, most of the cells with a round morphology were attached to all three surfaces (Figure 7). At 6 h after seeding, the osteoblasts showed different degrees of spreading. Some diffuse actin and vinculin were expressed inside the less-spreading cells while in the elongated ones a weak developing actin network could be remarked. Furthermore, discrete punctiform signals for vinculin have been found at cell periphery. This vinculin labeling pattern suggests formation of focal contacts between plasma membrane and extracellular proteins adsorbed to materials' surfaces. At a later time point (24 h), fluorescence analysis revealed typical appearance of osteoblast cells with well-expressed stress fibers oriented parallel to one another and to the long axis of the cell and punctiform vinculin signals at the termini of actin microfilament bundles. At all-time points, no significant differences in terms of cell spreading, cytoskeleton organization, and focal contact formation could be observed between analyzed samples. Overall, our results demonstrate that the bare and nitrided Ti-23Nb-0.7Ta-2Zr-0.5N represent suitable substrates for cell adhesion and cell proliferation, as well.

## 4. Conclusions

The thickness of the native passive film existing on the bare Ti-23Nb-0.7Ta-2Zr-0.5N alloy surface is 6.5 ± 0.5 nm. For the nitrided alloy, the thickness of the formed layer could not be determined because it is a very thick coating. The XPS high resolution spectra differ very much referring to the bare and nitrided Ti-23Nb-0.7Ta-2Zr-0.5N alloy. The massive presence of the nitrogen that reacts with the surface and with the bulk of the alloy was observed for the nitrided alloy. The nitrogen is also observed in depth in the range of microns, forming nitrides and oxynitrides and diminishing the thickness of the native passive film under 5 nm. Oxygen has a lower concentration correlated to the much higher nitrogen atom concentration if compared with the bare alloy. Thus, the nitriding treatment strongly influences both the composition and properties of the surface, of the substrate and of the bulk alloy.

SEM images revealed an etched-like texture, after nitration with the grain boundaries ranges between 200 nm and around 1 *μ*m, indicating that the N has penetrated inside the alloy grains. The EDX elemental analysis detected the alloy elements and nitrogen; for the nitrided alloy, there is obviously a decrease of N content from the surface towards the inside of the grains.

Polarization curves of the treated alloy in Ringer's solution shifted to left and to more positive potentials showing an ennobling of the corrosion and passivation potentials and a reduction of the passive current density; the passive current density values are maintained very constantly on the whole passive potential range, indicating the continuous thickening of the protective layers only by the ionic transfer inside these layers, in concordance with the high field mechanism. All electrochemical results confirmed the high chemical stability of the nitride and oxynitride coating and the superiority of the applied treatment. The values of the corrosion parameters ascertained the excellent corrosion resistance of the coated alloy in the real functional conditions from the human body.

Furthermore, cell culture experiments with MG63 osteoblast-like cell line proved that all studied biomaterials favor cell proliferation without showing any deleterious effect suggesting increased biocompatibility. Based on all these findings, nitrided Ti-23Nb-0.7Ta-2Zr-0.5N alloy demonstrated great potential to be used as future prosthesis material.

## Figures and Tables

**Table 1:** Atom relative concentrations (atom %) of the initial films existing on the bare and nitrided Ti-23Nb-0.7Ta-2Zr-0.5N alloy surface.

Alloy	Element relative concentrations (at.%)					
	Ti 2p	Nb	Ta 4f	Zr 3d	O 1s	N 1s
Bare	42.1	26.0	0.8	1.4	21.2	0.5
Nitrided	37.8	16.8	0.5	2.0	3.0	39.9

**Table 2:** Main electrochemical parameters for Ti and bare and nitrided Ti-23Nb-0.7Ta-2Zr-0.5N alloy in Ringer's solution of different pH values, at 37°C.

Material	*E*_corr_ (mV)	*E*_*p*_ (mV)	Δ*E*_*p*_ (mV)	|*E*_corr_ − *E*_*p*_| (mV)	*i*_*p*_ (*μ*A/cm^2^)
Ringer pH = 3.21					
Ti	−400	−200	>1000	200	25.1
Bare Ti-23Nb-0.7Ta-2Zr-0.5N	−350	−150	>1000	200	6.3
Nitrided Ti-23Nb-0.7Ta-2Zr-0.5N	−250	−100	>1000	150	3.2

Ringer pH = 7.58					
Ti	−320	−200	>1000	120	15.2
Bare Ti-23Nb-0.7Ta-2Zr-0.5N	−250	−150	>1000	100	2.3
Nitrided Ti-23Nb-0.7Ta-2Zr-0.5N	−150	−50	>1000	50	1.2

Ringer pH = 8.91					
Ti	−475	−275	>1000	200	18.3
Bare Ti-23Nb-0.7Ta-2Zr-0.5N	−350	−250	>1000	100	5.5
Nitrided Ti-23Nb-0.7Ta-2Zr-0.5N	−250	−150	>1000	100	3.3

**Table 3:** Main corrosion parameters for Ti and bare and nitrided Ti-23Nb-0.7Ta-2Zr-0.5N alloy in Ringer's solution of different pH values, at 37°C.

Material	*i*_corr_ (*μ*A/cm^2^)	*E* (%)	*V*_corr_ (*μ*m/Y)	Ion release (ng/cm^2^)	Class	*R*_*p*_ (kΩ cm^2^)	*P* (%)
Ringer pH = 3.21							
Ti	0.74	—	8.625	876.3	FS	11.3	—
Bare Ti-23Nb-0.7Ta-2Zr-0.5N	0.097	—	0.868	88.19	PS	257.9	—
Nitrided Ti-23Nb-0.7Ta-2Zr-0.5N	0.0085	91.24	0.076	7.72	PS	784.6	32.83

Ringer pH = 7.58							
Ti	0.724	—	8.326	845.9	FS	18.2	—
Bare Ti-23Nb-0.7Ta-2Zr-0.5N	0.031	—	0.277	28.2	PS	351.3	—
Nitrided Ti-23Nb-0.7Ta-2Zr-0.5N	0.0026	91.61	0.023	2.55	PS	957.2	36.70

Ringer pH = 8.91							
Ti	1.186	—	13.7	1391.9	S	13.9	—
Bare Ti-23Nb-0.7Ta-2Zr-0.5N	0.092	—	0.823	83.62	PS	275.8	—
Nitrided Ti-23Nb-0.7Ta-2Zr-0.5N	0.0081	91.20	0.073	7.42	PS	772.1	35.72

## References

[1] Geetha M., Singh A. K., Asokamani R., Gogia A. K. (2009). Ti based biomaterials, the ultimate choice for orthopaedic implants—a review. *Progress in Materials Science*.

[2] Huiskes R., Weinans H., van Rietbergen B. (1992). The relationship between stress shielding and bone resorption around total hip stems and the effects of flexible materials. *Clinical Orthopaedics and Related Research*.

[3] Hallab N. J., Vermes C., Messina C., Roebuck K. A., Glant T. T., Jacobs J. J. (2002). Concentration- and composition-dependent effects of metal ions on human MG-63 osteoblasts. *Journal of Biomedical Materials Research*.

[4] Linter C. M. (1985). Neuropsychiatric aspects of trace elements. *British Journal of Hospital Medicine*.

[5] Kim H. Y., Ikehara Y., Kim J. I., Hosoda H., Miyazaki S. (2006). Martensitic transformation, shape memory effect and superelasticity of Ti-Nb binary alloys. *Acta Materialia*.

[6] Kim H. Y., Sasaki T., Okutsu K. (2006). Texture and shape memory behavior of Ti-22Nb-6Ta alloy. *Acta Materialia*.

[7] Bertrand E., Gloriant T., Gordin D. M. (2010). Synthesis and characterisation of a new superelastic Ti-25Ta-25Nb biomedical alloy. *Journal of the Mechanical Behavior of Biomedical Materials*.

[8] Bertrand E., Castany P., Gloriant T. (2013). Investigation of the martensitic transformation and the damping behavior of a superelastic Ti-Ta-Nb alloy. *Acta Materialia*.

[9] Kim J. I., Kim H. Y., Inamura T., Hosoda H., Miyazaki S. (2005). Shape memory characteristics of Ti-22Nb-(2-8)Zr(at.%) biomedical alloys. *Materials Science and Engineering A*.

[10] Sun F., Hao Y. L., Nowak S., Gloriant T., Laheurte P., Prima F. (2011). A thermo-mechanical treatment to improve the superelastic performances of biomedical Ti–26Nb and Ti–20Nb–6Zr (at.%) alloys. *Journal of the Mechanical Behavior of Biomedical Materials*.

[11] Meng Q., Guo S., Liu Q., Hu L., Zhao X., Meng Q. (2014). A *β*-type TiNbZr alloy with low modulus and high strength for biomedical applications. *Progress in Natural Science: Materials International*.

[12] Al-Zain Y., Kim H. Y., Hosoda H., Nam T. H., Miyazaki S. (2010). Shape memory properties of Ti-Nb-Mo biomedical alloys. *Acta Materialia*.

[13] Wang B. L., Zheng Y. F., Zhao L. C. (2008). Effects of Sn content on the microstructure, phase constitution and shape memory effect of Ti-Nb-Sn alloys. *Materials Science and Engineering A*.

[14] Tahara M., Kim H. Y., Hosoda H., Miyazaki S. (2009). Shape memory effect and cyclic deformation behavior of Ti-Nb-N alloys. *Functional Materials Letters*.

[15] Ramarolahy A., Castany P., Prima F., Laheurte P., Péron I., Gloriant T. (2012). Microstructure and mechanical behavior of superelastic Ti–24Nb–0.5O and Ti–24Nb–0.5N biomedical alloys. *Journal of the Mechanical Behavior of Biomedical Materials*.

[16] Hao Y. L., Li S. J., Sun S. Y., Yang R. (2006). Effect of Zr and Sn on Young's modulus and superelasticity of Ti–Nb-based alloys. *Materials Science and Engineering A*.

[17] Yang Y., Castany P., Cornen M., Thibon I., Prima F., Gloriant T. (2014). Texture investigation of the superelastic Ti-24Nb-4Zr-8Sn alloy. *Journal of Alloys and Compounds*.

[18] Fu J., Yamamoto A., Kim H. Y., Hosoda H., Miyazaki S. (2015). Novel Ti-base superelastic alloys with large recovery strain and excellent biocompatibility. *Acta Biomaterialia*.

[19] Ijaz M. F., Kim H. Y., Hosoda H., Miyazaki S. (2014). Effect of Sn addition on stress hysteresis and superelastic properties of a Ti-15Nb-3Mo alloy. *Scripta Materialia*.

[20] Huang R., Zhuang H., Han Y. (2014). Second-phase-dependent grain refinement in Ti-25Nb-3Mo-3Zr-2Sn alloy and its enhanced osteoblast response. *Materials Science and Engineering C*.

[21] Xue P., Li Y., Li K., Zhang D., Zhou C. (2015). Superelasticity, corrosion resistance and biocompatibility of the Ti-19Zr-10Nb-1Fe alloy. *Materials Science and Engineering C*.

[22] Zhecheva A., Sha W., Malinov S., Long A. (2005). Enhancing the microstructure and properties of titanium alloys through nitriding and other surface engineering methods. *Surface and Coatings Technology*.

[23] Zhecheva A., Malinov S., Sha W. (2006). Titanium alloys after surface gas nitriding. *Surface and Coatings Technology*.

[24] Gordin D. M., Thibon I., Guillou A., Cornen M., Gloriant T. (2010). Microstructural characterization of nitrided beta Ti-Mo alloys at 1400°C. *Materials Characterization*.

[25] Li H., Cui Z., Li Z., Zhu S., Yang X. (2014). Surface modification by gas nitriding for improving cavitation erosion resistance of CP-Ti. *Applied Surface Science*.

[26] Januszewicz B., Klimek L. (2010). Nitriding of titanium and Ti6Al4V alloy in ammonia gas under low pressure. *Materials Science and Technology*.

[27] Sathish S., Geetha M., Pandey N. D., Richard C., Asokamani R. (2010). Studies on the corrosion and wear behavior of the laser nitrided biomedical titanium and its alloys. *Materials Science and Engineering C*.

[28] Höche D., Schaaf P. (2011). Laser nitriding: investigations on the model system TiN. A review. *Heat and Mass Transfer*.

[29] Vora H. D., Rajamure R. S., Dahotre S. N., Ho Y.-H., Banerjee R., Dahotre N. B. (2014). Integrated experimental and theoretical approach for corrosion and wear evaluation of laser surface nitrided, Ti–6Al–4V biomaterial in physiological solution. *Journal of the Mechanical Behavior of Biomedical Materials*.

[30] Mishra S. C., Nayak B. B., Mohanty B. C., Mills B. (2003). Surface nitriding of titanium in arc plasma. *Journal of Materials Processing Technology*.

[31] Fouquet V., Pichon L., Drouet M., Straboni A. (2004). Plasma assisted nitridation of Ti-6Al-4V. *Applied Surface Science*.

[32] El-Hossary F. M., Negm N. Z., Khalil S. M., Raaif M. (2005). Effect of continuous and cyclic Rf plasma processing time on titanium surface. *Applied Surface Science*.

[33] Sun J., Tong W. P., Zuo L., Wang Z. B. (2013). Low-temperature plasma nitriding of titanium layer on Ti/Al clad sheet. *Materials and Design*.

[34] Gordin D. M., Guillou A., Thibon I., Bohn M., Ansel D., Gloriant T. (2008). Duplex nitriding treatment of a beta-metastable Ti_94_Mo_6_ alloy for biomedical applications. *Journal of Alloys and Compounds*.

[35] Sarró M. I., Moreno D. A., Ranninger C., King E., Ruiz J. (2006). Influence of gas nitriding of Ti6Al4V alloy at high temperature on the adhesion of *Staphylococcus aureus*. *Surface and Coatings Technology*.

[36] Annunziata M., Oliva A., Basile M. A. (2011). The effects of titanium nitride-coating on the topographic and biological features of TPS implant surfaces. *Journal of Dentistry*.

[37] Lin N., Huang X., Zhang X., Fan A., Qin L., Tang B. (2012). In vitro assessments on bacterial adhesion and corrosion performance of TiN coating on Ti6Al4V titanium alloy synthesized by multi-arc ion plating. *Applied Surface Science*.

[38] Lin N., Huang X., Zou J. (2012). Effects of plasma nitriding and multiple arc ion plating TiN coating on bacterial adhesion of commercial pure titanium via in vitro investigations. *Surface and Coatings Technology*.

[39] Zhao X., Yan D., Li S., Lu C. (2011). The effect of heat treatment on the electrochemical corrosion behavior of reactive plasma-sprayed TiN coatings. *Applied Surface Science*.

[40] Kim W.-G., Choe H.-C. (2012). Effects of TiN coating on the corrosion of nanostructured Ti-30Ta-xZr alloys for dental implants. *Applied Surface Science*.

[41] Singh R., Chowdhury S. G., Tiwari S. K., Dahotre N. B. (2008). Laser surface processing of Ti6Al4V in gaseous nitrogen: corrosion performance in physiological solution. *Journal of Materials Science: Materials in Medicine*.

[42] Gordin D. M., Gloriant T., Chane-Pane V. (2012). Surface characterization and biocompatibility of titanium alloys implanted with nitrogen by Hardion+ technology. *Journal of Materials Science: Materials in Medicine*.

[43] Hashimoto M., Kashiwagi K., Kitaoka S. (2011). A nitrogen doped TiO_2_ layer on Ti metal for the enhanced formation of apatite. *Journal of Materials Science: Materials in Medicine*.

[44] Yildiz F., Yetim A. F., Alsaran A., Çelik A. (2008). Plasma nitriding behavior of Ti6Al4V orthopedic alloy. *Surface and Coatings Technology*.

[45] Savonov G. S., Ueda M., Oliveira R. M., Otani C. (2011). Electrochemical behavior of the Ti6Al4V alloy implanted by nitrogen PIII. *Surface and Coatings Technology*.

[46] Pham V.-H., Yook S.-W., Lee E.-J. (2011). Deposition of TiN films on Co-Cr for improving mechanical properties and biocompatibility using reactive DC sputtering. *Journal of Materials Science: Materials in Medicine*.

[47] Huang H.-H., Hsu C.-H., Pan S.-J., He J.-L., Chen C.-C., Lee T.-L. (2005). Corrosion and cell adhesion behavior of TiN-coated and ion-nitrided titanium for dental applications. *Applied Surface Science*.

[48] Jang H. W., Lee H.-J., Ha J.-Y., Kim K.-H., Kwon T.-Y. (2011). Surface characteristics and osteoblast cell response on TiN- and TiAlN-coated Ti implant. *Biomedical Engineering Letters*.

[49] Ion R., Gordin D.-M., Mitran V. (2014). In vitro bio-functional performances of the novel superelastic beta-type Ti-23Nb-0.7Ta-2Zr-0.5N alloy. *Materials Science and Engineering C*.

[50] Roman D., Bernardi J. C., Boeira C. D. (2012). Nanomechanical and electrochemical properties of ZrN coated NiTi shape memory alloy. *Surface and Coatings Technology*.

[51] Caicedo J. C., Zambrano G., Aperador W., Escobar-Alarcon L., Camps E. (2011). Mechanical and electrochemical characterization of vanadium nitride (VN) thin films. *Applied Surface Science*.

[52] Calderon Moreno J. M., Vasilescu E., Drob P. (2013). Surface and electrochemical characterization of a new ternary titanium based alloy behaviour in electrolytes of varying pH. *Corrosion Science*.

[53] Gordin D. M., Ion R., Vasilescu C., Drob S. I., Cimpean A., Gloriant T. (2014). Potentiality of the ‘Gum Metal’ titanium-based alloy for biomedical applications. *Materials Science and Engineering C*.

[54] Mareci D., Chelariu R., Gordin D.-M., Ungureanu G., Gloriant T. (2009). Comparative corrosion study of Ti-Ta alloys for dental applications. *Acta Biomaterialia*.

[55] Tato W., Landolt D. (1998). Electrochemical determination of the porosity of single and duplex PVD coatings of titanium and titanium nitride on brass. *Journal of the Electrochemical Society*.

[56] Cai Z., Nakajima H., Woldu M., Berglund A., Bergman M., Okabe T. (1999). In vitro corrosion resistance of titanium made using different fabrication methods. *Biomaterials*.

[57] Van Noort R. (1987). Titanium: the implant material of today. *Journal of Materials Science*.

[58] Cimpean A., Mitran V., Ciofrangeanu C. M. (2012). Osteoblast cell behavior on the new beta-type Ti-25Ta-25Nb alloy. *Materials Science and Engineering C*.

[59] Moulder J. F., Stckle W. F., Sobol P. E., Bomben K. D. (1995). *Handbook of X-Ray Photoelectron Spectroscopy*.

[60] Naumkin A. V., Kraut-Vass A., Gaarenstroom S. W., Powel C. J. (2012). NIST X-ray photoelectron spectroscopy database. *NIST Standard Reference Database 20 Version 4.1*.

[61] Baer D. R., Engelhard M. H., Lea A. S. (2010). Comparison of the sputter rates of oxide films relative to the sputter rate of SiO_2_. *Journal of Vacuum Science & Technology A*.

[62] Lohrengel M. M. (1993). Thin anodic oxide layers on aluminium and other valve metals: high field regime. *Materials Science and Engineering R: Reports*.

[63] Park C. H., Lee C. S., Kim Y.-J., Jang J.-H., Suh J.-Y., Park J.-W. (2011). Improved pre-osteoblast response and mechanical compatibility of ultrafine-grained Ti-13Nb-13Zr alloy. *Clinical Oral Implants Research*.

[64] Humphries J. D., Wang P., Streuli C., Geiger B., Humphries M. J., Ballestrem C. (2007). Vinculin controls focal adhesion formation by direct interactions with talin and actin. *The Journal of Cell Biology*.

